# Life cycle assessment of a direct air capture and storage plant in Ireland

**DOI:** 10.1038/s41598-023-44709-z

**Published:** 2023-10-25

**Authors:** Daniel Casaban, Elena Tsalaporta

**Affiliations:** 1 Discipline of Process and Chemical Engineering, School of Engineering, Environmental Research Institute, University College Cork, Cork, Republic of Ireland; 2 Discipline of Process and Chemical Engineering, School of Engineering, Environmental Research Institute, University College Cork, Room 329, Food Science Building, 1 College Rd, Cork, T12 TP07 Republic of Ireland

**Keywords:** Climate sciences, Climate-change mitigation, Climate-change policy, Environmental impact, Sustainability

## Abstract

Despite the efforts to transition to a low carbon economy, greenhouse gas emissions are surging to critical levels. Carbon dioxide removals (CDR) methods, such as direct air capture (DAC), have been gaining substantial public attention in the last few years. DAC is essential in curbing CO_2_ concentrations and achieving climate targets. It is said that DAC can be deployed at anywhere, but a throughout life cycle assessment (LCA) is imperative to prove its viability. Therefore, this paper aims to explore the feasibility of constructing a 1 $\text{MT}_{{\text{CO}}_{2}}$ plant in Ireland, using Kinsale and Corrib gas fields as storage points. The results showed that the country is an ideal candidate for scaling up this emerging industry. The efficiency is primarily influenced by the construction of the pipeline section, given a reliable sources of heat and electricity. The study highlights the significant impact of distances to the storage points on feasibility, favouring counties near of the gas fields. In conclusion, Ireland has the potential to establish its own DAC industry.

## Introduction

Numerous countries have formulated strategies to tackle climate change; however, the world continues in track towards surpassing 2 °C threshold above pre-industrial levels^1^. Global Greenhouse Gas (GHGs) emissions rebounded after 2020, reaching a staggering 52.8 $\text{Gt}_{\text{CO}_\text{2}e}$^2^, which is affecting our society^3^. Low carbon emitting technologies (LCET) are anticipated to serve as a bridge towards a net-zero economy^4^. In line with this, the Intergovernmental Panel on Climate Change (IPCC) has recommended the adoption of Carbon Dioxide Removals (CDRs), such as Direct Air Capture (DAC), to remove six GtCO_2_ annually by 2050^5^. These CDR methods are considered essential in addressing the ongoing climate crisis.

Over the last two decades, scientists have devoted significant research efforts on investigating adsorption and absorption techniques to extract CO_2_ from the atmosphere^6^. In adsorption processes, ambient air is passed through a solid material with a high affinity for retaining CO_2_, while absorption involves the use of an aqueous solution^7^. Notably, one of the key advantages of absorption over adsorption is the capacity to operate under continuously^8^. Conversely, adsorption excels in its ability to perform the process with reduced water and energy needs^7^. Both methods have been demonstrated feasible, leading to the construction of the first industrial plants by certain companies, with ongoing developments in this field^9,10^.

In order to execute the process and mitigate the most severe effects of climate change, a substantial portion of the captured CO_2_ must be stored^11^. Depleted oil and gas fields emerge as one of the most effective solutions for CO_2_ storage^12^. Lessons and insights gleaned from prior experiences demonstrated the feasibility of this approach^13^. Additionally, deep saline aquifers have been investigated as geological formations suitable for CO_2_ storage^14^. These reservoirs are estimated to possess larger storage capacities compared to oil and gas fields^15^. However, the storage capacity of these geological formations hinges on the specific characteristics of the aquifer and the complexity of the storage operation^16^.

Structural storage involves the underground pumping of CO_2_; because of its lighter density compared to water, the gas ascends through the porous rock until reaches the top of the geological formation, where it becomes trapped and securely sealed^17^. In the case of saline aquifers, CO_2_ storage is achieved by a process known as saline trapping. In this case, rocks act as a sponge, where the injected CO_2_ remains trapped within the pore spaces^18^. To enhance its stability, CO_2_ can be dissolved into saline water to increase its density and causing it to sink to the lowermost regions of the rock formation, thereby permanently entrapping the CO_2_^19^. Moreover, CO_2_ dissolved in saline water exhibits reduced acidity and can react with minerals in adjacent rocks, facilitating the formation of new minerals^20^. Currently, numerous projects and experiments are being investigated in Iceland, where they are storing CO_2_ in basalt rocks^21^. Furthermore, some studies suggested converting CO_2_ into cement through mineral carbonation using Ca^+^ and Mg^+^ rich materials^22^.

Thus, the DAC industry is poised to grow its capacity in the current decade. As previously mentioned, the primary objective of this emerging industry is the permanent storage of CO_2_. Nevertheless, certain sectors are expected to keep emitting GHGs in the coming years In response, the captured CO_2_ can be re-utilised to offset emissions from agriculture or be synthesised into alternative fuels for aviation or chemical applications^23^. To comprehensively evaluate the efficiency of this process and its environmental implications, a Life Cycle Assessment (LCA) is essential for gaining insights and know the effectiveness of the overall process^24,25^.

Climeworks is expected to open a new DAC plant in Iceland, taking advantage of the surplus of geothermal energy^26^. Their LCA study highlighted carbon dioxide removal efficiencies of 90%, affirming the efficacy of their process^27^. The adaptability to build a DAC plant in any location depends on the accessibility to a rich renewable energy source^28^. Moreover, it is imperative to recognise other factors during its construction and operation that can affect the performance of the CO_2_ capture. As Terlouw et al. stressed, the transportation and storage of CO_2_ can have detrimental impacts to the environment^29^. The necessary infrastructure for gas transport, the drilling and injection operations, and the possible CO_2_ leakage, can collectively elevate the energy demands, thereby contributing to a higher carbon footprint^30^.

The Republic of Ireland has the EU's fastest-growing economy, yet its national development plans did not take into account DAC solutions^23^. Ireland overarching objectives is to reduce its carbon emissions by transitioning from fossil gas to renewable energy sources within its electricity grid^31^. Additionally, the government aspires to reduce transport emissions by introducing one million electric vehicles by 2030, while heat pumps are expected to replace oil and gas in the residential sector. These transformative initiatives have the potential significantly reduce the country’s reliance on fossil fuels. Nonetheless, it is noteworthy that no LCA study has been conducted to highlight the importance of DAC process in future scenario projections. 

From this perspective, the objective of this LCA study is to investigate various potential sites for a prospective DAC plant in Ireland. The country has two potential storage sites the examination of which will provide insights to understand the environmental impacts and the energy requirements associated with the capture and storage processes^23^. A precedent LCA from Climeworks underscores the necessity of a low carbon source to power the plant^32^. While Ireland lacks geothermal energy resources, it boast consistently robust wind speeds, offering the potential to generate enough energy to exceed the domestic demand^31^.

## Methodology

During this study, SimaPro was the software utilised for the comprehensive assessment of materials, energy requirements, and environmental impacts throughout the construction and operation of a DAC plant in Ireland. It is important to remark that some of the system boundaries were influenced by the data confidentiality inherent to this emerging technology. The DAC process technique for this study is based on the Climeworks plant, specifically, the Temperature-Vacuum Swing Adsorption process. Therefore this reliance constitutes a notable limitation within the context of this work. To achieve a more detailed analysis, it is crucial to examinate all the engineering processes behind of a real DAC plant.

Similar to many industrial processes, the operation of a DAC plant will be influenced by the energy source employed. In order to evaluate the climate change impacts, we utilised the EF 3.0 Method, which incorporates the Global Warming Potential to 100 years (GWP100) as an indicative metric for quantifying the GHGs in terms of CO_2_ equivalent^33^. Thus, the primary goal of this study is to quantify the kg of CO_2_ emitted per ton of CO_2_ removed, encompassing all phases of the process: construction, operation, and storage. In order to assess the carbon efficiency, we established three thresholds at 25%, 50% and 75%. Additionally, we followed the methodology and guidance provided by Terlouw to expose our study case^34^. In this context, the authors made assumptions to approximate the impacts from the construction of the DAC plant: CO_2_ collectors, process unit, steel tanks and a maintenance hall, for control the process.

### Construction of a DAC plant

The Collector boxes are composed of materials such as plastic, steel, insulation, and fan, which is remarkably similar to the Life Cycle Inventory (LCI) of a typical passenger vehicle^35^. In account of this similarity, we adopted their approximation as a basis for our assessment, and we selected a 1200 kg compact size petrol/natural gas car as a proxy for the collectors. As stated by Climeworks, 80 collector boxes have the capacity to capture 4 ktCO_2_ per year in the plant located at Iceland^10^. Each collector measures 2 m in width, height and length, resulting in a total volume of 640 m^3^, with a corresponding density of 110 kg/m^3^^32^. These assumptions provided a base for the evaluation of the collector boxes during this study.

In addition to the collector boxes, a passenger vehicle was used as a representative approximation for the process unit. A DAC unit is comprised by two containers, each of which accommodates 9 collector boxes. These containers have a length of 12 m and a width and height of 2 m. Collectively, the total volume of the process unit is estimated to be 50 m^3^. In the case of the DAC plant from Climeworks called ORCA, it needs 9 process units, amounting a total volume of 450 m^3^ in order to capture 4$\text{kt}_{\text{CO}_\text{2}}$ per year^10^. Additionally, the infrastructure requires the inclusion of one steel tank per 18 collector boxes. These steel tanks have a diameter, height, and thickness of 2 m, 6 m and 0.02 respectively.

The construction of the DAC plant requires the transformation of grassland into an industrial area, overlaying it with concrete for the foundations^36^. Climeworks reports a requirement of 90 m^2^ for the DAC plant located at Hinwil^37^. Terlouw et al., linearly scale the land use for both, the ORCA plant and a prospective 100 kt plant^34^. Their findings indicated that, for the 4$\text{kt}_{\text{CO}_\text{2}}$ and 100 $\text{kt}_{\text{CO}_\text{2}}$ plants, concrete layers of 400 m^2^ and 5000 m^2^, respectively, each with a thickness of 1 m are imperative. Moreover, to ensure the structural integrity, the concrete should be reinforced with 120 kg steel/m^3^^27^.

**Figure 1:**
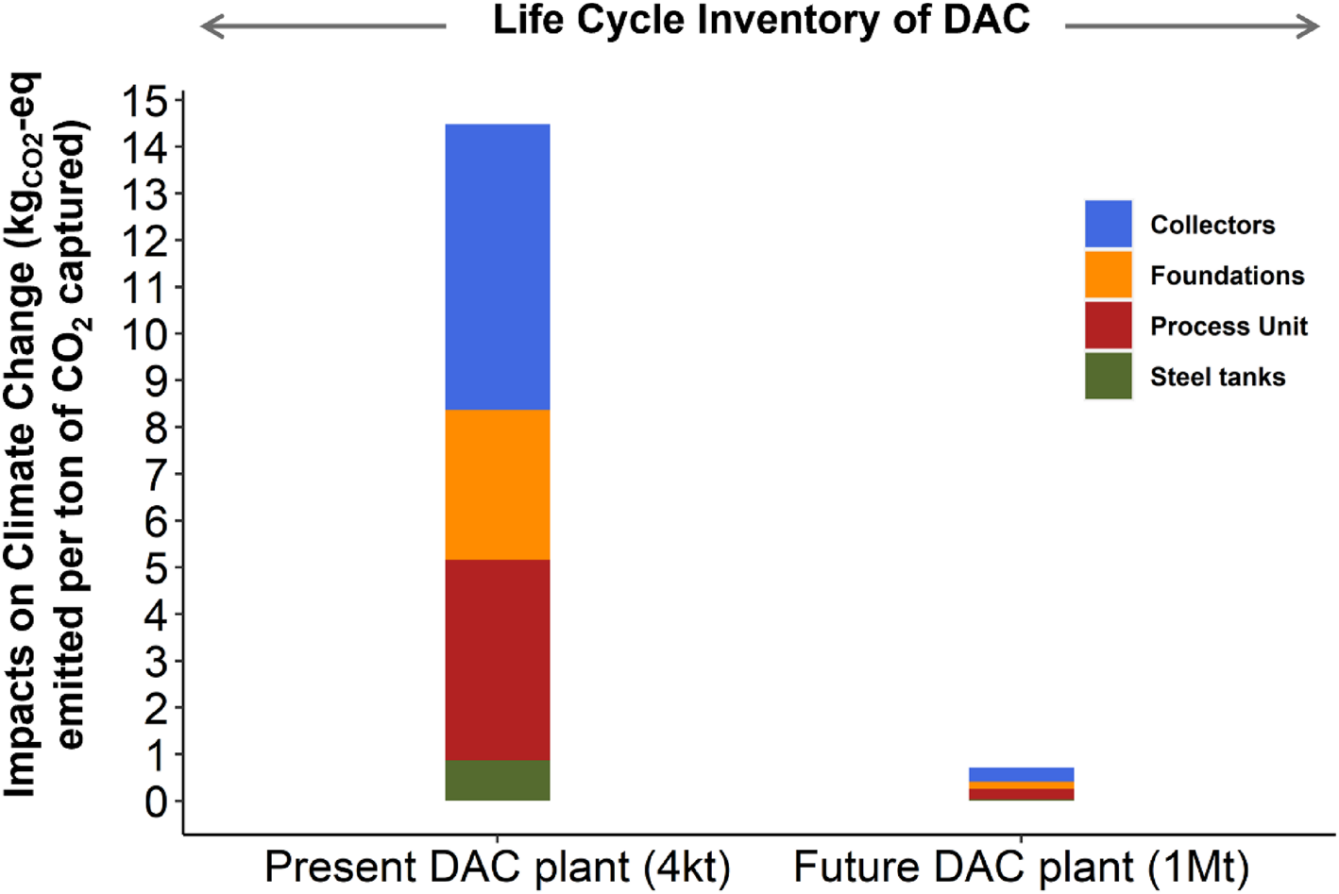
Impacts on climate change from the life cycle inventory for a DAC plant with of 4 kt and megaton capacity.

While the exact dimensions of the maintenance hall remain unclear^36^, Terlouw et al. estimated that this structure should occupy 75% of the total surface area. To validate the accuracy of our results (Fig. 1), a comparative analysis was conducted with the data from Climeworks and Terlouw. In their research, they reported figures of 14.4 and 13.5 kg of CO_2_ emitted per ton capture for the 4$\text{kt}_{\text{CO}_\text{2}}$ plant, and 5.8 and 6.7 kg of CO_2_ emitted per ton capture for the 100 $\text{kt}_{\text{CO}_\text{2}}$ plant. In our study, we obtained similar results indicating 14.5 kg of CO_2_ per ton captured for the 4$\text{kt}_{\text{CO}_\text{2}}$ and 6.3 kg of CO_2_ per ton captured for the 100$\text{kt}_{\text{CO}_\text{2}}$ Therefore, we scaled our results to adapt these figures for the requirements of our megaton-capacity plant. Notably, future installations are anticipated to require less quantities of materials and land use, driven by advancement in the field such as improved techniques or the development of novel sorbents^38^.

### Operation of a DAC plant

**Figure 2:**
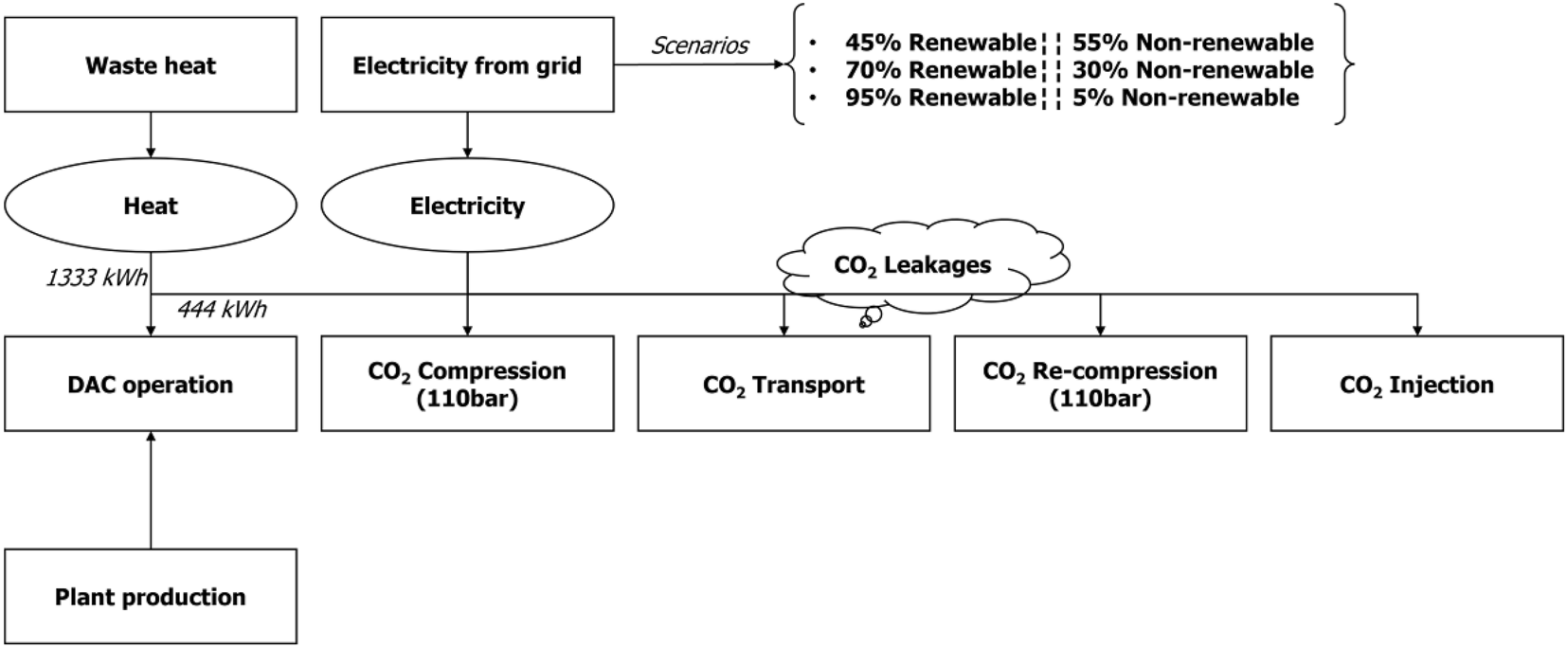
Diagram of a DAC + S according to every scenario. Scenario 1: 55% of the energy comes from fossil origin and 45% from renewable. Scenario 2: 30% of the energy comes from fossil origin and 70% from renewable. Scenario 3: 5% of the energy comes from fossil origin and 95% from renewable.

According to Climeworks, their plant currently requires of 500 kWh of electricity and 1500 kWh of heat to operate^37^. However, recent studies suggested that ongoing improvements in the process will reduce the energy needs to 444 kWh of electricity and 1333 kWh of heat per ton of CO_2_ captured (Fig. 2)^39^. In order to meet the heat demands, our assumption is that the necessary energy for the process can be sourced from a chemical industry or from a waste incineration plant, such as the one located in Dublin^40^. In 2020, approximately 3.2 million tonnes of municipal waste were generated, with 41% of it being transformed into energy^41^. The availability of a source of waste heat has the potential to significantly reduce emissions and environmental impacts associated with the process^42^. For the electricity needs, we hypothesised that the renewable energy predominantly originates from wind sources, while non-renewable is sourced from gas plants within the island. This scenario is premised on the expectation that the country’s sole coal plant of the country will cease its operations in the near future^43^. However, should Moneypoint coal plant continue to operate beyond 2030, it would result in an increment of the CO_2_ emissions from the electricity requirements, consequently amplifying the associated environmental impacts. Thus, the scenarios from our study are based on the future development plans of the country^44^.

### CO_2_ storage

According to Hendriks et al.^45^, there is a method to estimate the energy needs for the injection step. To transport the CO_2_, the gas should be compressed from 1 to 110 bar in a four-step centrifugal compression^46^. If the capacity of the plant is of about 1 $\text{Mt}_{\text{CO}_\text{2}}$ per year with a Flh value of 8160, the mass flow of the process is 34 $\text{kg}_{\text{CO}_\text{2}}$/s or 122.4 $\text{t}_{\text{CO}_\text{2}}$/h. All of these parameters are considered in Eq. (1), where Ce is a constant with a value of 87.85 kj/kg.

1 $$E={C}_{el}*ln\left(\frac{{P}_{outlet}}{{P}_{inlet}}\right)*F$$

The results indicated that a total of 115 kWh of energy is needed for the combined compression stages. Moreover, an additional input of 12 kWh is required to inject the CO_2_ to a depth of at least 2000 m underground. Consequently, the cumulative energy input from the transport and storage process amounts approximately 130 kWh per ton of CO_2_ removed. This energy will be provided depending on the specific energy mix associated to the established scenarios.

In alignment with Terlouw approach^34^, we considered the potential fugitive emissions of CO_2_ during its transportation within of the pipelines, categorised as a medium baseline scenario. Moreover, Holloway et al. developed a method to estimate the loss of CO_2_ during its transmission^47^. According to their research, the CO_2_ emissions rate is 1.66 times that of the CH_4_-emission rate. Thus, this rate is multiplied by the annual distribution emission factor of CH_4_ to calculate the estimate losses of CO_2_ for the different DAC plants across the country (Table 1)^48^. It is noteworthy that the maximum depth of Kinsale gas field is 1000 m, while Corrib can reach depths of up to 3000 m (Table 2). However, storage depths around 2000 m have negligible effects on the LCA^49^. Therefore, the potential CO_2_ leakage from the injection wells is deemed insignificant in the case of Kinsale^50^.

**Table 1:** Leakage emissions from transporting CO_2_ to Kinsale and Corrib via pipeline.

DAC plant	Distance to Kinsale	Distance to Corrib	Leakage to Kinsale	Leakage to Corrib
	Gas field (km)	Gas field (km)	Gas field ($\text{kg}_{\text{CO}_\text{2}}$)	Gas field ($\text{kg}_{\text{CO}_\text{2}}$)
Castlebar	316	128	45	18.23
Donegal	393	195	55.96	27.77
Dublin	260	323	37.02	46
Kinsale	74	336	10.54	47.85
Tipperary	150	281	21.36	40.01
Wexford	171	377	24.35	53.69

**Table 2:** Characteristics from some of the practical and effective storage basin locations in Ireland.

Site name	Location	Storage type	Status	Storage capacity ($\text{Mt}_{\text{CO}_\text{2}}$)	Reservoir type	Reservoir depth (m)
Kinsale	Celtic Sea Basin	Gas field	Depleted	350	Triassic sandstone	800
Spanish Point	Porcupine Basin	Gas field	Depleted	120	Jurassic Voligian sandstones	–
Corrib	Northwest Basin	Gas field	Operational	6950	Lower Cretaceous sandstone	2000
East Irish Sea Basin	Irish Sea Basin	Aquifer	–	630	Triassic sandstone	900
East Irish Sea	Irish Sea Basin	Oil & gas field	Depleted	1050	Triassic sandstone	900
Kish Bank	Central Irish Sea Basin	Aquifer	–	267	Sherwood sandstone	1750
Lough Neagh	Northern Ireland	Aquifer	–	1940	Sherwood sandstone	1300

**Figure 3:**
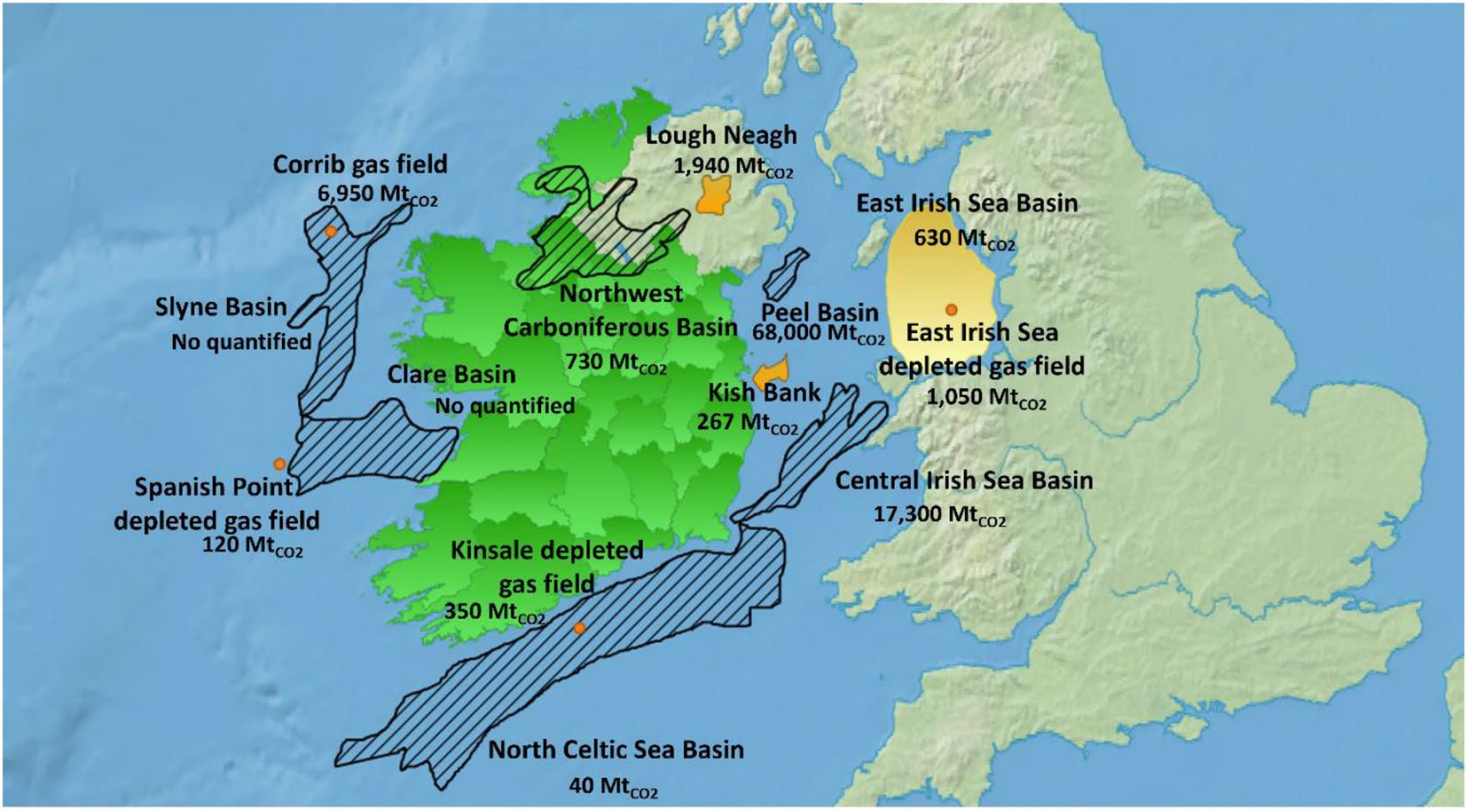
Most important storage points near of Ireland. The points represent the oil & gas fields. The hashed basins represent the capacity in theory, and the coloured ones represent the effective CO_2_ storage capacity. Map generated by QGIS 3.28.3 Geographic Information System: http://www.qgis.org.

While there are numerous potential CO_2_ storage locations in close proximity to Ireland, with a capacity to store 93,115 $\text{Mt}_{\text{CO}_\text{2}}$ (Fig. 3), it is important to remark that only a relative small portion of these sites can be considered practical or effective at the moment (1505 $\text{Mt}_{\text{CO}_\text{2}}$)^51,52^. Therefore, due to the existing pipeline infrastructure, there is enough data to support the selection of Kinsale's gas field as favourable location for CO_2_ storage^53^.

Moreover, the depletion of the Corrib gas field in the coming years presents a dual challenge and opportunity for Ireland. It poses a potential issue for national energy security, but simultaneously offers an opportunity to transition towards renewable energy sources of energy as well as bolstering a local DAC industry^23,54^. While the prospect of storing the abated CO_2_ in other locations remains viable, it is imperative to address both political and technical challenges before its implementation. For instance, the UK government has plans to utilise the Irish Sea Basin to store CO_2_^55^. The basin particularly located at Lough Neagh, is one of the most important wetland habitats in Europe and provides multiple ecosystem services^56^. It is important to emphasise that the data pertaining to Lough Neagh only considers the effective capacity of a small area in the basin, and uncertainties exist regarding the thickness of the seal layer, which is essential for an effective CO_2_ storage^51^. Therefore, given its significant ecological importance, any consideration of Lough Neagh Basin for CO_2_ storage will require an exhaustive and meticulous study to assess potential impacts on this natural ecosystem.

The concern of seismic activity during the CO_2_ storage has been a recurring topic of discussion and has been widely explored in the past^57^. In 2026, Scotland is going to build the first DAC plant with a megaton capacity^9^. This particularly plant relies in absorption processes, but it is worth noting that solid sorbent DAC plants are rapidly progressing and may soon achieve similar capacities^26,58^. In light of this, we have assumed that the plant of this current study has a capacity to remove 1 $\text{Mt}_{\text{CO}_\text{2}}$ per year. Furthermore, this study suggested six potential locations for a DAC plant deployment across Ireland: Kinsale, Tipperary, Wexford, Dublin, Castlebar, Donegal.

## Results and discussion

The displayed results serve to demonstrate the feasibility of establishing a DAC plant in the country. It is noteworthy that solid sorbent processes only require modest levels of energy consumption. Currently, the DAC facility utilising this approach has demonstrated a maximum capture capacity of 36 $\text{kt}_{\text{CO}_\text{2}}$ per year^26^. Yet, it is important to remark the significant progress witnessed over the past 2 years, during which the capacity for CO_2_ capture is one magnitude order higher^10^. It is anticipated that within the next decade, these plants will achieve the megaton-scale capacity^9^. At the moment, the only DAC plants constructed are strategically located areas endowed with a high presence of renewable energy resources, and the storage points are situated close to the capture plant. Ireland, in alignment with sustainable goals, is moving towards low carbon energy sources energy, and in the near future, the country expect to meet 70% of its electricity demand by renewable sources. Recent developments, such as the successful first major auction to implement offshore wind power, mark a significant milestone of a promising future within the power sector (Shortt, R. 2023). Nevertheless, it is imperative to remark that renewable energy have certain limitations, and addressing emissions from sectors that are finding difficulties need CDR and carbon capture methods.

**Figure 4:**
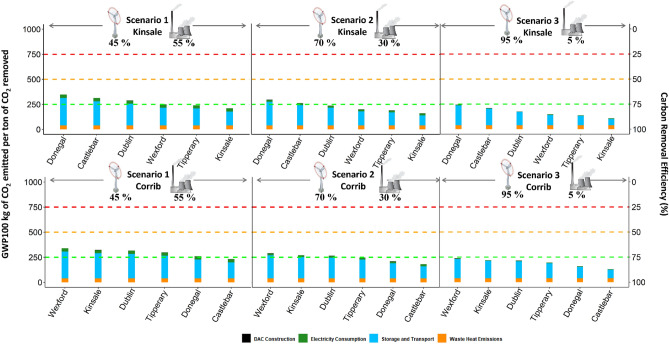
Life cycle emissions per ton of CO_2_ removed from a DAC plant of one megaton capacity constructed different locations according to each scenario and storage location. The share at the second y-axis represents the efficiency of the process. The colours of the bar display the contribution of each process. In the storage and transport is included the infrastructure requirements and construction impacts.

In our study, conducted across various scenarios within both study cases, it is important to remark that emissions did not surpass the half ton of CO_2_ emitted per ton captured as illustrated in Fig. 4. The construction phase of the plant has a minimum impact during its operational lifetime. Across all the considered, it was possible to note a correlation between the source of electricity and efficiency of the plant. Long-term projections, particularly those in close proximity to Corrib and Kinsale gas fields, indicate a promising trajectory towards enhanced efficiency. As the wind power sector matures in the country, supplying the bulk of the energy demands, it is possible to reach efficiency levels 87% or 89%. Nevertheless, it was revealed that the consequences on the environment from the assembly of the pipeline section had to be taken into account for the future planning of the plant. The energy requisites for both, the operation of the plant and CO_2_ transport, were presupposed according to the future development plans^44^. However, it is pertinent to underscore that the emissions from electricity needs can vary depending on the source of electricity. A concurrent study remarks the significance of this important factor to another study and how the carbon efficiency can drop to 10% if the electricity of the grid comes from fossil sources^59^. Within their analysis, it was evident that a mere 18.9% energy from renewable sources can affect the whole process. This assertion finds similarities with the work from Madhu et al., who contend that a low-carbon energy source (70 g CO_2_eq kWh^−1^), can elevate the carbon efficiency of the DAC + S system to nearly 86%^60^.

The integration of heat derived from waste plants was an important factor in reducing the carbon footprint of the process. Nevertheless, it is imperative to note that these emissions were included in our analysis to comprehensively assess the impacts on the overall process. In instances where waste heat is not accessible, the emissions incurred during the operation can diminish the capture efficiency^34^. In that case, the consideration of a heat pump becomes pertinent^27^. A coefficient of performance of 2.51 is sufficient to meet the operational requirements of the plant, falling within the typical range of heat pumps in the market^61^. Thus, a throughout study is vital to identify neighbouring industries capable of provide a rich source of waste heat to support the operational demands of the plant.

**Figure 5:**
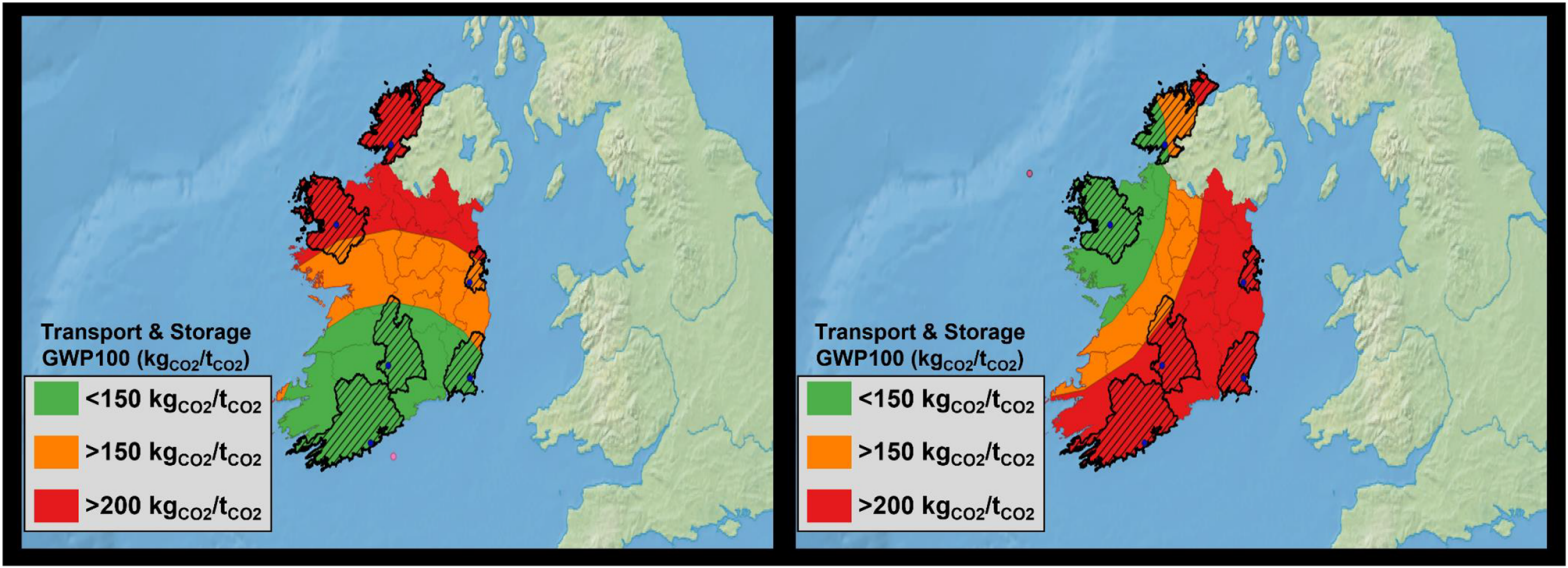
GHGs emissions in kg of CO_2eq_ per ton of CO_2_ transported to the storage place from the DAC plant. During the transport of CO_2_ is included the leakages from Table 1. Maps generated by QGIS 3.28.3 Geographic Information System: http://www.qgis.org.

The emissions associated with the transportation of CO_2_ are linked to the construction of the pipeline section, with emissions increasing proportionally to the distance from the capture point to the storage place. Additionally, the energy requirements for the compression and delivery of the CO_2_ also increase with greater distances, necessitating an additional compression step for distances exceeding 200 km. In the context of both study cases in scenario 1, it becomes evident that the storage and transport processes can reduce the carbon removal efficiency up to 25%. This effect can be visualised in Fig. 4, underscoring the importance of the emissions during the construction of the piping system. Consequently, to account the emissions caused during the construction of the transportation infrastructure, we segmented it into three diverse areas based on its carbon footprint (Fig. 5). It is important to remark that this study possesses a limitation and assumes a direct, linear route for CO_2_ transport from the plant to the storage point. Nonetheless, leveraging the current infrastructure can offer opportunities to reduce emissions and render the transport of CO_2_ viable for other regions. Other studies have suggested this solution, with the costs associated with CO_2_ transportation representing a small fraction of the overall DAC emissions^62^.

The elevated temperatures and pressures at the depths of the reservoirs induce changes in the characteristics of the CO_2_, facilitating the storage a larger volume. At a depth of 800 m, CO_2_ reaches the critical point where both its temperature and pressure excess 31 °C and 73 atm, respectively. At this state, the volume of gas contracts to approximately 0.32 times that of its surface state. Given the ideal conditions for building a DAC plant in the country, a megaton scale plant is capable of storing 48.54 million of m^3^ over its operational lifetime. In light of these considerations, 17 plants of 1 $\text{Mt}_{\text{CO}_\text{2}}$ capacity will be sufficient to fill the Kinsale depleted gas field. If we consider the natural carbon sinks, the accumulated emissions from 1990 to 2023 from Ireland are almost 500 $\text{Mt}_{\text{CO}_\text{2}e}$^23^. Regardless of the progress in the field or the prospected emergence of future gigaton plants by 2050, this approach shows the potential of DAC to contribute to the mitigation of half of the accumulated emissions. As previously mentioned, DAC remains in its nascent stages of development, yet there are many similarities with the renewable energy sector in the 1990s^63^. In order to achieve the desired levels of carbon dioxide removal, it becomes imperative explore materials and methods applicable under atmospheric conditions^6^. It is important to highlight that the presence of moisture in the ambient air can pose challenges, reducing the process efficiency and increasing the electricity consumption for dehumidification purposes.

Nevertheless, the findings of this study establish Ireland as an ideal candidate for the deployment of a DAC plant in the near future. Taking advantage of the wind and a reliable source of heat from waste, promises to substantially reduce the energy costs associated to the DAC plant operation, specifically, solid sorbent-based technologies^64^. However, this represents the optimal scenario, and not every region within the country may meet the requisite conditions. Our analysis revealed that the most important factor lies in the transport and storage process. Counties close to Kinsale and Corrib gas fields have an advantage in contrast with the others, as they do not need the same infrastructure requirements to transport the CO_2_. This presents a potential catalyst for the local economy. However, there are viable alternatives for the midland regions of the country, offering opportunities for equitable deployment of DAC facilities.

Deploy a DAC in plant in midlands counties may present challenges when it comes to storing CO_2_ in Kinsale or Corrib, yet the Northwest Carbonaceous can store an effective quantity of 730 $\text{Mt}_{\text{CO}_\text{2}}$. A more viable approach would involve the construction of DAC plants in Limerick or Dublin. Despite these counties are relative distant from the storage points examined of this study, they have other nearest locations where store the CO_2_. In the vicinity of Dublin lies the Kish Bank basin (Table 2), with an effective capacity for storing up to 270 $\text{Mt}_{\text{CO}_\text{2}}$. Thus, the CO_2_ can be delivered to regional nearby reservoirs, such as the Central Irish Sea Basin or on the other hand, to the North Sea through shipping, which is the biggest storage point in Europe^65^. This strategic shift could enhance the viability of different locations for a DAC plant due to the construction of a pipeline infrastructure will not be a requisite^66^ and therefore, reduce the associated emissions.

## Conclusions

Because of its robust economic growth and strategic geographical positioning, Ireland has significant potential in the emerging DAC industry. Notably, the agricultural sector plays a crucial role in the country’s economy. In the near future, it is imperative for this sector to reduce its emissions by a substantial 25%. Therefore, there will persist “hard-to-abate” emissions that requires effective mitigation measures. A reliable DAC industry can be instrumental in closing the carbon cycle by addressing these residual emissions. If the process is powered by renewable energy sources, part of the CO_2_ can be converted into e-fuels for the aviation and shipping industry, as for example the production of e-methanol and e-kerosene. These approaches hold the potential to significantly reduce the emissions associated with storage and transport, although they may incur increased energy costs, specifically associated to the use of an electrolyse. Future studies will scrutinise this aspect in order to determine its viability.

Therefore, Ireland can find itself in an advantageous position to become pioneers in the fields of renewable energy and CDR. While renewable energy is expected to expand in the coming decade, CDR and carbon capture lag behind. It is crucial to highlight that no single technology can solve the climate crisis. Renewables face its own challenges, such as energy transmission and storage. Simultaneously, there is a crucial need to curtail the costs associated with the capture of CO_2_ from certain CDRs and carbon capture techniques. It is important to stress that CDRs, including DAC, should complement emissions reductions efforts and serve to neutralise any residual emissions from sectors with difficulties to decarbonise. If these technologies are integrated with other climate policies, in combination with renewable energy, can collectively fulfil their respective roles.

Encouragingly, last years have witnessed a shift in perspective. Renewable energy is booming, and CDR and carbon capture technologies are garnering increased recognition and support, with new initiatives emerging. Numerous DAC plants and planned projects are on the horizon this decade, with the expectation of providing valuable insights into this critical technology. Therefore, the availability of data proved to be a significant limiting factor during our analysis, making our LCA reliant on prior studies.

In an effort to minimise the carbon footprint of the operation, we considered that the megaton DAC plant would be powered by waste heat and renewable energy. Our study focused primarily on the utilisation of the depleted gas fields of Kinsale and the future depleted gas field at Corrib. According to every scenario Castlebar and Kinsale emerged as highly favourable locations for the construction of a DAC plant, owing to their proximity to the storage location. Most of the emissions from the process are attributed to the transport and storage of the CO_2_. Nonetheless, DAC plants can be established in various locations throughout the country. By levering the existing gas transportation network, counties such as Waterford have the opportunity to establish a DAC plant. Additionally, close to Dublin and in the Irish Sea, particularly the Kish Bank Basin and East Irish Basin, offers the substantial storage capacity of 1050 $\text{Mt}_{\text{CO}_\text{2}}$^e^. Counties such as Donegal can explore the potential of the onshore Northwest Carboniferous Basin, which is estimated to store 730 $\text{Mt}_{\text{CO}_\text{2}}$^e^. To facilitate these initiatives, comprehensive exploration operations and environmental risk assessments must be conducted at the known and other un-quantified basins, such as the Clare Basin located in Limerick.

## Data Availability

The datasets used and/or analysed during the current study available from the corresponding author on reasonable request.
